# Automated deep bottleneck residual 82-layered architecture with Bayesian optimization for the classification of brain and common maternal fetal ultrasound planes

**DOI:** 10.3389/fmed.2023.1330218

**Published:** 2023-12-20

**Authors:** Fatima Rauf, Muhammad Attique Khan, Ali Kashif Bashir, Kiran Jabeen, Ameer Hamza, Ahmed Ibrahim Alzahrani, Nasser Alalwan, Anum Masood

**Affiliations:** ^1^Department of Computer Science, HITEC University, Taxila, Pakistan; ^2^Department of Computing and Mathematics, Manchester Metropolitan University, Manchester, United Kingdom; ^3^Computer Science Department, Community College, King Saud University, Riyadh, Saudi Arabia; ^4^Department of Circulation and Medical Imaging, Norwegian University of Science and Technology, Trondheim, Norway; ^5^Institute of Neurosciences and Medicine (INM), Forschungszentrum Jülich, Jülich, Germany

**Keywords:** maternal fetal, biomedical imaging, deep learning, residual architecture, bottleneck layers, optimization

## Abstract

Despite a worldwide decline in maternal mortality over the past two decades, a significant gap persists between low- and high-income countries, with 94% of maternal mortality concentrated in low and middle-income nations. Ultrasound serves as a prevalent diagnostic tool in prenatal care for monitoring fetal growth and development. Nevertheless, acquiring standard fetal ultrasound planes with accurate anatomical structures proves challenging and time-intensive, even for skilled sonographers. Therefore, for determining common maternal fetuses from ultrasound images, an automated computer-aided diagnostic (CAD) system is required. A new residual bottleneck mechanism-based deep learning architecture has been proposed that includes 82 layers deep. The proposed architecture has added three residual blocks, each including two highway paths and one skip connection. In addition, a convolutional layer has been added of size 3 × 3 before each residual block. In the training process, several hyper parameters have been initialized using Bayesian optimization (BO) rather than manual initialization. Deep features are extracted from the average pooling layer and performed the classification. In the classification process, an increase occurred in the computational time; therefore, we proposed an improved search-based moth flame optimization algorithm for optimal feature selection. The data is then classified using neural network classifiers based on the selected features. The experimental phase involved the analysis of ultrasound images, specifically focusing on fetal brain and common maternal fetal images. The proposed method achieved 78.5% and 79.4% accuracy for brain fetal planes and common maternal fetal planes. Comparison with several pre-trained neural nets and state-of-the-art (SOTA) optimization algorithms shows improved accuracy.

## 1 Introduction

The brain is the key organ of our body, and as such, it influences every aspect of the body. Brain disorders bring on multiple diseases (1). In this way, a mother’s healthy lifestyle affects her child’s brain development. The trajectory of the organism across the lifespan and succeeding lifelong functions are determined by fetal and newborn brain development (2). The story of the human fetal brain is an intricate journey marked by substantial alterations in size, configuration, and advancement, following a distinctive spatiotemporal pattern (3). Irregular fetal brain development can have significant short-term and long-term impacts on the newborn (4). Therefore, precise quantitative evaluation of fetal brain growth is crucial for the early detection of developmental disorders (5). Furthermore, early detection of these abnormalities might enhance the accuracy of the diagnosis and follow-up preparation (6).

A standard medical tool for non-invasively assessing and monitoring the state of the developing brain *in utero* is magnetic resonance imaging (MRI) (7). Ultrasound also serves as a standard method for tracking the progress of a developing human fetus, offering valuable insights into its growth and general well-being (8). Throughout the different stages of gestation, ultrasound examinations are conducted to verify pregnancy, assess its position, condition, dimensions, growth rate, alignment, and gestational age, detect possible congenital disabilities and complications, and gather various other details crucial for ensuring the fetus’s healthy development and delivery (9). Fetal brain abnormalities can often be found with ultrasound. Doctor’s lack of familiarity with complicated brain anatomy and pathology, obsolete technology, an improper fetal head position, an early or late gestational age, and maternal obesity all reduce the detection rate. To become experts, doctors must have years of experience (10).

The researchers have employed computer-aided diagnosis techniques, including advanced tools based on deep learning, to automate the measurement of fetal body parts (11). Due to their excellent prediction accuracy and human-level performance across several medical imaging applications, deep learning models have gained prominence (12). Deep learning consists of layers of nonlinear information processing within a hierarchical structure designed for extracting features, analyzing patterns, and classifying data (13). In the realm of fetal ultrasound image analysis, deep learning, notably convolutional neural networks (CNNs), has become increasingly pivotal over the past few decades (14). Its application has significantly enhanced decision support for medical professionals by providing advanced capabilities in the interpretation of fetal ultrasound images. As a result, a substantial body of literature is dedicated to this field (15).

Shinde et al. (16) combined the information of deep learning features with the traditional machine learning methods for classifying fetal brain abnormalities using MRI scans. The Random Forest (RF) classifier is employed for machine learning, whereas a pre-trained architecture has been employed for deep learning. The experimental findings from the DNN + RF model were compared with those from the DNN + SVM and plain DNN frameworks. It demonstrates that the presented method performed well for the DNN + RF framework with an accuracy of 94 and 87% for training and validation, respectively. Kumar et al. (17) presented a cloud environment to discover and categorize fetal brain disorders automatically. The system’s main goal was to perfect the art of fetal brain abnormality detection while eliminating or drastically cutting down on time, expense, and accuracy. The YOLO v4 architecture was used to identify the fetal brain with its orientation. The presented method obtained an accuracy of 97.27%.

Qu et al. (18) introduced a distinctive CNN architecture to autonomously discern six fetal brain standard planes (FBSPs) from non-standard planes. The supplementary differential feature maps within this framework were formulated by extracting differential operators from the feature maps in the original CNN. A significant benefit of differential convolution maps was their ability to analyze the directional pattern of pixels and their surroundings utilizing additional calculations. The differential CNN performed well and obtained an improved accuracy of 92.93%. Płotka et al. (19) developed an end-to-end multi-task neural network named Fetal Net. The presented method analyzes spatiotemporal fetal ultrasound scan videos by means of integrated modules and an attention mechanism. Its objective is to measure, classify, and determine several fetal body parts at the same time once. In order to achieve effective localization of scan planes, researchers used an attention mechanism combined with a stacked module. Ye et al. (20) introduced a deep neural network for classifying five fetal head ultrasound planes, including trans ventricular plane (TV), trans thalamic plane (TT), transcerebellar plane (TC), coronal view of eyes (Eyes), coronal view of the nose. This model also identified non-standard fetal head ultrasound images effectively. Ruowei et al. (21) designed two primary methods based on deep convolutional neural networks to recognize fetal brains automatically. One method used a deep convolutional neural network (CNN), while the other employed CNN-based domain transfer learning.

Shankar et al. (22) suggested deep learning (DL) method for computing three key fetal brain biometric measurements from 2D ultrasound images of the transcerebellar plane (TC) through automated caliper placement. Di et al. (23) presented a regression convolutional neural network (CNN) architecture for fetal brain classification from Ultrasound images. Singh et al. (24) used a pre-trained ResNet-50 architecture to classify fetal ultrasound images of crown-to-rump length (CRL). The model employed a skip connection strategy to develop a deeper network with task-specific hyper parameters. The presented architecture performed well on the selected dataset.

**Figure 1:**
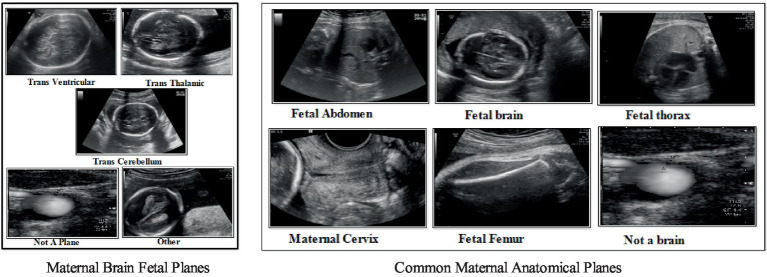
A few sample images of common maternal fetal and fetal brain ultrasound images.

The studies discussed above are focused on CNN architectures to compute the improved recognition accuracy of maternal-fetal (25). In this work, our core aim is to explore the strength of deep learning architectures for classifying common maternal fetuses, including brain classes. The ultrasound images dataset has been utilized in this work for the validation process, with a selection of sample images depicted in Figure 1. In this figure, it is noted that there are similar characteristics of the maternal brain fetal planes with a high chance of false negative rate. Similarly, the common maternal anatomical planes are similar and difficult to recognize with simple machine learning or deep learning models. There are challenges in classifying maternal-fetal complexities using traditional methods, which include shape, texture, and point features, because of their intricate and similar textures. Therefore, the deep learning architecture based on the bottleneck mechanism can work more effectively to classify the maternal-fetal tasks. We introduced an innovative deep learning architecture that leverages residual bottleneck blocks and minimizes pooling layers, capitalizing on the benefits of the deep bottleneck mechanism. The primary contributions of our work are outlined as follows:

- Proposed a new CNN architecture based on the residual-bottleneck mechanism that includes three residual blocks (two paths and one bypass). One skip connection and traditional layers path include three convolutional layers of 1 × 1, 3 × 3, and 1 × 1 filter sizes.
- Bayesian optimization is performed to initialize the hyper parameters of the designed residual bottleneck CNN architecture: the learning rate, epochs, momentum, batch size, and L2-regularization factor.
- The average pooling layer is used to extract deep features, and an improved moth flame optimization algorithm is presented with an updated search space for the best feature selection. Several neural network classifiers are used to classify the selected features.
- A detailed ablation study has been performed to compare the performance of the proposed architecture with several other combinations of feature selection algorithms and neural nets. Also, a comparison has been conducted with few recent published methods.

The article’s remaining sections are arranged as follows: proposed methodology is presented under section 2. Results and detailed ablation study discussed in Section 3. Section 4 concludes the manuscript that followed the references.

## 2 Proposed methodology

**Figure 2:**
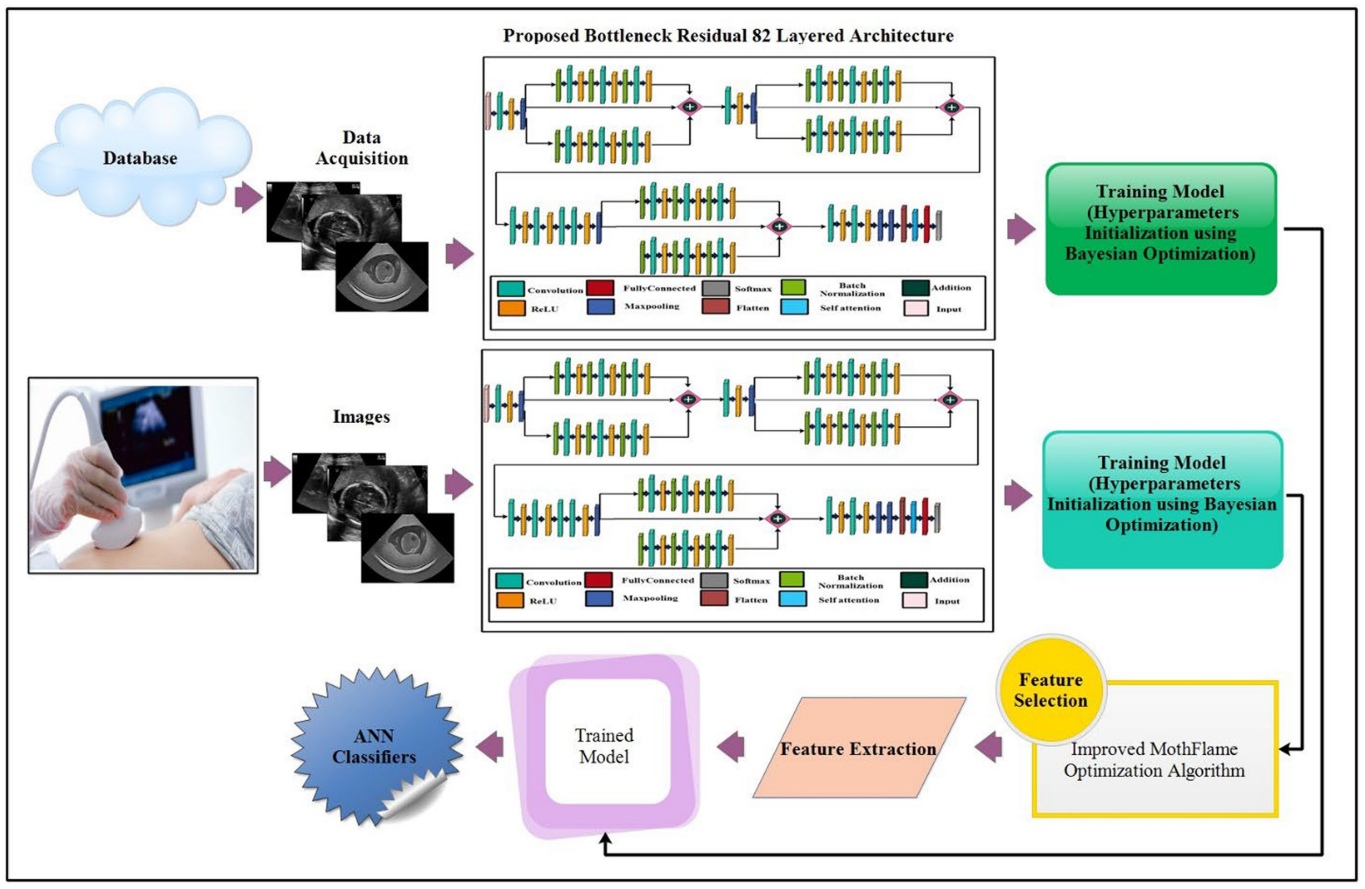
Proposed architecture of brain fetal planes and common maternal fetal planes classification using bottleneck residual CNN.

The proposed maternal brain fetal planes and common maternal fetal classification architecture have been presented in this section. The proposed framework consists of two phases: first, classify the fetal brain using ultrasound images, and second, fetal general characteristics using ultrasound images. Figure 2 illustrates the proposed framework. In the proposed framework, a new model has been proposed based on three residual blocks that include a bottleneck mechanism. The proposed model consists of 3 residual blocks that include several layers in a bottleneck fashion. In the training phase, hyper parameters have been initialized using Bayesian optimization. The self-attention layer has been selected and activation is performed for deep feature extraction. The deep features extracted are fine-tuned through an enhanced moth flame optimization algorithm that incorporates a position update mechanism. Neural network classifiers are finally used in the last stage to perform the final classification.

### 2.1 Datasets

This study uses a publicly available dataset downloaded from the cloud and stored in a local cloud (Google Drive). The dataset contains two types: Fetal Brain and common maternal fetal classes. The details of the datasets are given below.

#### 2.1.1 Fetal brain classes

In the fetal brain, five classes have been included: trans cerebellum (TC), trans thalamic (TT), trans ventricular (TV), not a brain, and others. A few examples of images are shown in Figure 1. Each class has a different number of images, as presented in Table 1. The table illustrates that the highest count of images in a single class is 9,308 (not a brain), while the lowest count is 143.

**Table 1:** A summary of Fetal Brain and common maternal fetal images of the selected dataset.

Fetal brain dataset			Common maternal fetal dataset		
Class	#Images	Training/Testing	Class	#Images	Training/Testing
Trans cerebellum	714	357/357	Fetal abdomen	1,422	711/711
Trans thalamic	1,638	819/819	Fetal thorax	1,718	858/857
Trans ventricular	597	299/298	Fetal brain	3,092	1,546/1,546
Not a brain	9,308	4,654/4,654	Not a brain	4,213	21,066/21,065
Other	143	72/71	Maternal cervix	1,626	813/813
			Fetal femur	2,080	1,040/1,040

#### 2.1.2 Common maternal fetal

In the common maternal fetal dataset, six classes have been included: fetal abdomen, fetal brain, fetal femur, fetal thorax, maternal cervix, and not a brain. A few examples of images are shown in Figure 1. A summary of the number of images has been added under Table 1. This table shows that the number of images is in the range of 1,422–4,213. Therefore, it seems there is a class imbalance issue for both types. However, we did not perform augmentation and tried to resolve this issue by proposing a new deep model with complex structures that can easily classify the maternal-fetal classes.

### 2.2 Proposed 82 layered bottleneck architecture

**Figure 3:**
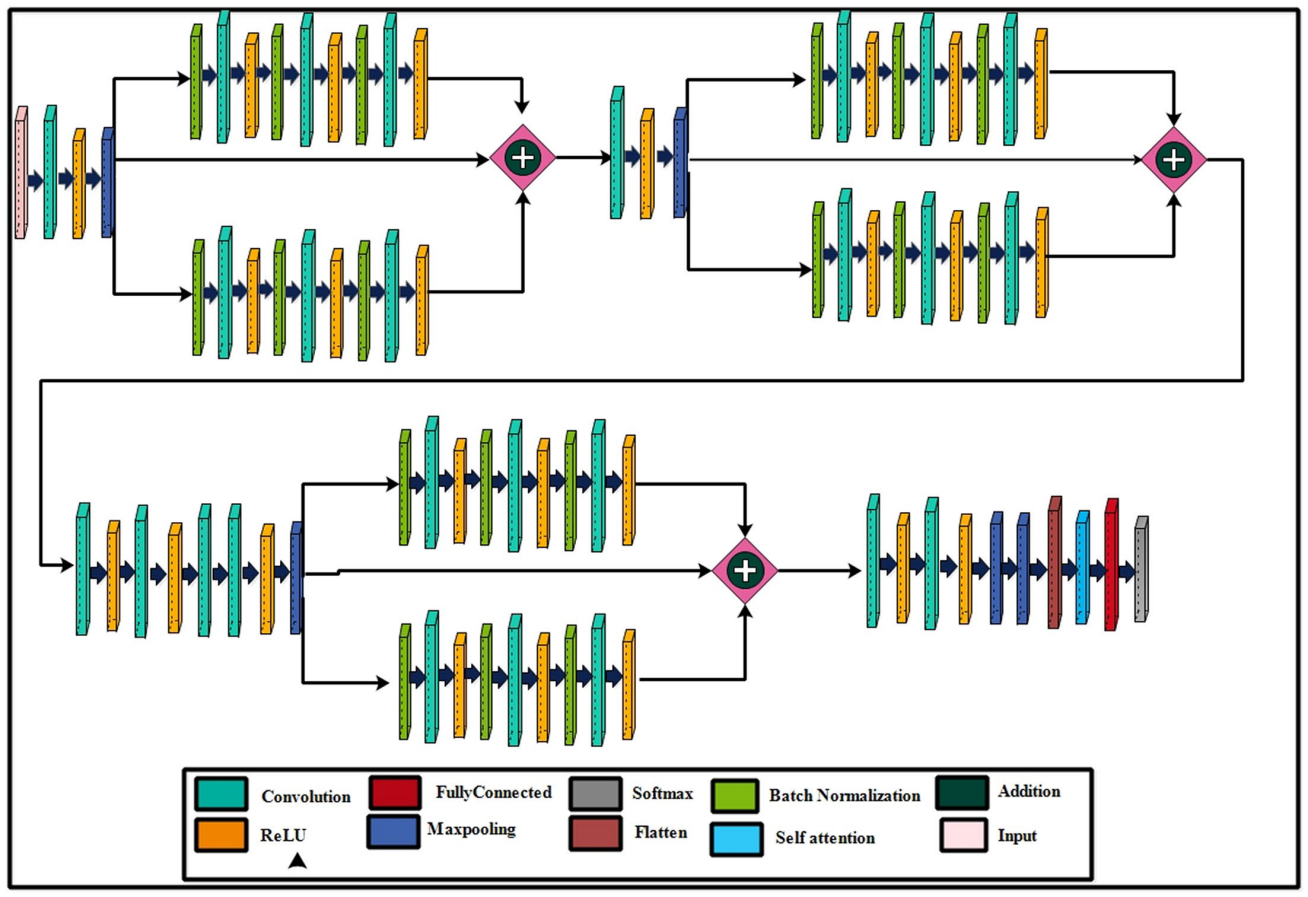
A visual architecture of the proposed 82-layered residual bottleneck CNN architecture.

The parallel residual blocks have been consist of two paths with different convolutional operations, and the filter sizes may vary across paths. These blocks are instrumental in capturing and processing information at different levels of abstraction in parallel, contributing to the overall depth and expressiveness of the model. A new architecture has been proposed in this work based on the concepts of residual blocks, bottleneck mechanisms, and hyper parameters optimization. The proposed architecture is shown in Figure 3. This figure shows the initial input layer of dimension 224 × 224 with a depth of 3. The model consist of total 26 convolutional layers. The subsequent layer is the first convolutional layer, which has a depth of 32 and employs a 3 × 3 convolutional filter size and stride of 2. Following each convolutional layer, a Rectified Linear Unit (ReLU) layer is added that is succeeded by a max-pooling layer featuring a 3 × 3 filter size and a stride of 1.

#### 2.2.1 First parallel bottleneck residual block

This network’s first bottleneck residual block is in parallel and shares a consistent layer pattern, totaling nine layers each. Each path of this block consists of batch normalization, convolutional, and ReLU layers. In the first block, a batch normalization layer with 32 channels is followed by a convolutional layer with a depth 128, a 1 × 1 filter size, and a stride of 1, concluding with a ReLU activation layer. The second block involves batch normalization with 128 channels, a convolutional layer with a depth of 512, a 3 × 3 filter size, and a stride of 1, followed by a ReLU layer. The third block encompasses batch normalization with 512 channels, a convolutional layer with a depth of 32, a 1 × 1 filter size, and a stride of 1, ending with a ReLU activation layer. A first addition layer connects these residual blocks to other layers.

#### 2.2.2 Intermediate layers 1

Following this, several additional layers are inserted within the network before the next parallel residual block has been added. A convolutional layer with a depth size 128 has been added, whereas the filter size is 3 × 3 and a stride of 2. After that, a ReLU activation layer was added, followed by the second max-pooling layer with a 3 × 3 filter size and a stride of 1.

#### 2.2.3 Second parallel bottleneck residual block

The second bottleneck residual block consists of two paths and one skip connection. Each path contains a total of nine layers, with each set consisting of batch normalization, convolutional, and ReLU layers. This block’s depth and filter sizes are similar to block 1. The main reason for the similar filter sizes of this block is to get deeper information on the processed images. After this, an additional layer has been added that connects the weights of both paths and skips the connection.

#### 2.2.4 Intermediate layers 2

Two sets of convolutional + ReLu layers have been added following these blocks. The first convolutional layer has a depth size of 128, while the second has a depth size of 256. The filter size of both convolutional layers has a 1 × 1 filter and maintains a stride of 2. Subsequently, two consecutive convolutional layers with depths of 64 and 512 have been added, having a 1 × 1 filter size and stride 2. After that, a max-pooling layer of filter size 3 × 3 and stride of 1 for downsampling has been added.

#### 2.2.5 Last bottleneck residual block

The last residual bottleneck block consists of similar layers like residual blocks 1 and 2. This block’s depth sizes are 256, 512, and 1,024. The filter size of the first convolutional is 1 × 1, the second layer is 3 × 3, and the last layer is 1 × 1, respectively. In addition, batch normalization and max pooling layer have been added in this block to speed up the training process and downsampling. Hence, nine layers have been added to each path, and finally, the additional layer with a skip connection.

#### 2.2.6 Final layers

**Figure 4:**
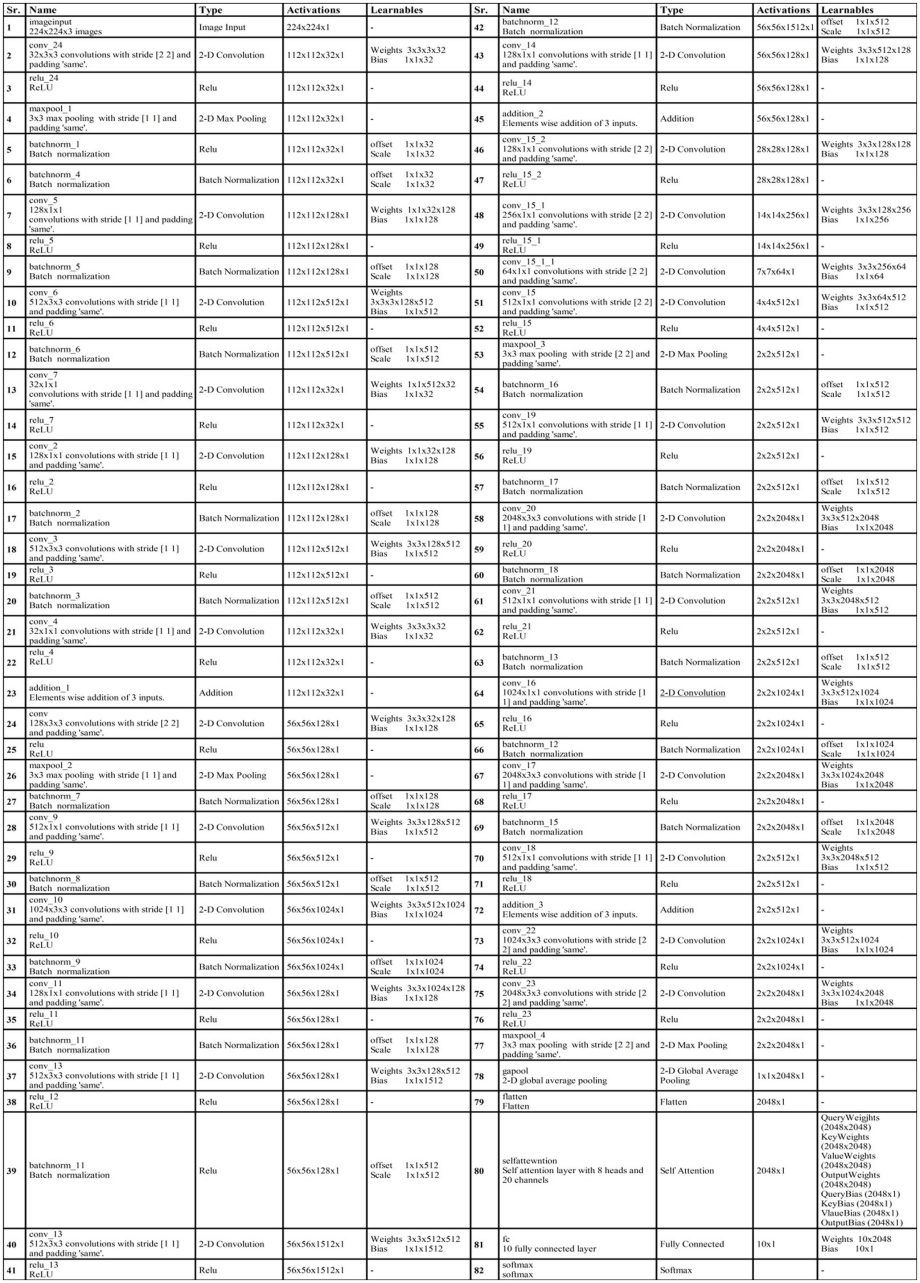
Detailed layered architecture of proposed bottleneck residual CNN architecture.

At the end of the model, a third addition layer connects these blocks with the remaining layers. Two convolutional layers are added, each followed by ReLU activation layers. The first convolutional layer has a depth of 1,024, whereas the second layer has a depth of 2048. The filter size of both layers is 3 × 3, and the stride value is 2. A max pool layer of filter size 3 × 3 and stride two has been added. Ultimately, a global average pooling layer is incorporated, succeeded by a fully connected layer, a Softmax layer, and a classification output layer. In addition, a tabular summary of the proposed architecture has been shown in Figure 4. The weights, filter sizes, stride, and total learnable have been added to this figure.

### 2.3 Proposed architecture training and features extraction

In the training procedure of the proposed architecture, several hyper parameters (HP) have been required to initialize. 78.5 M parameters have been trained for the proposed model. However, the manual selection of HP is always based on the expert person. Therefore, we tried to automate this system and implemented Bayesian optimization for the HP initialization. The following HP has been used in this work, optimized using BO: maximum epochs, initial learning rate, the mini-batch size, momentum, and L2-regularization factor.

#### 2.3.1 Bayesian optimization

An optimization problem known as “hyper parameter tuning” has an unidentified or “black-box” goal function. Using conventional optimization techniques like the Newton method or gradient descent is impossible. When handling this optimization problem, Bayesian optimization is a very successful optimization strategy (26). Using Bayesian formulas, it combines previous knowledge about unknown functions with sampled knowledge to compute posterior information. Finally, using this posterior knowledge, we may determine where the function gets its ideal value. The following are the main steps in the optimization process (27):

- The results of function F are updated using the posterior distribution taken by the Gaussian process.
- Selecting the ideal point using an acquisition function for the function F. The expected improvement is used as an acquisition function in this work.
- Recognizing the suggested sampling locations that were acquired by the acquisition function.
- Obtaining outcomes in the validation set using an objective function.
- Adding the most optimized sample points to the previously chosen data.
- Refreshing the model of the statistical Gaussian distribution.

**Figure 5:**
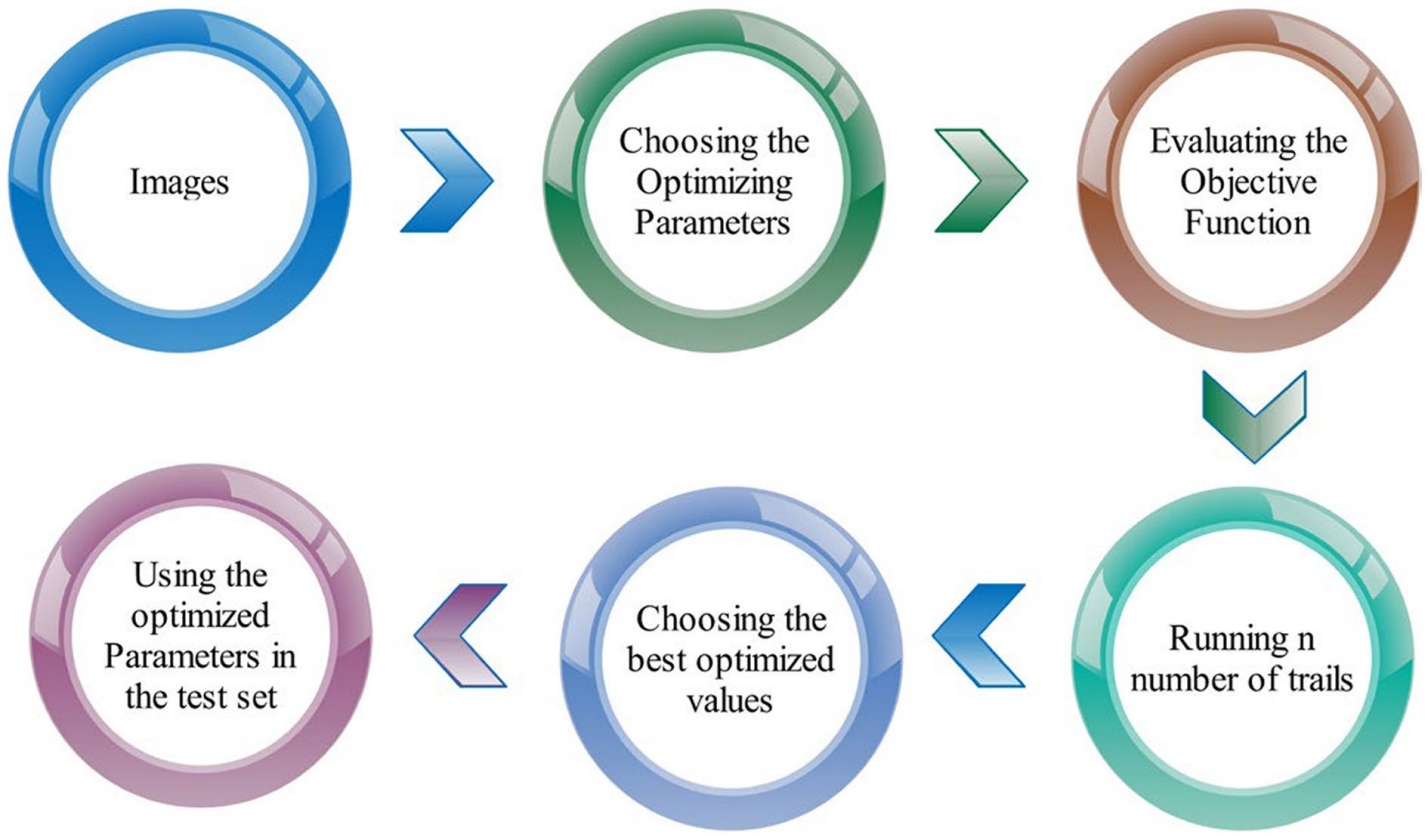
A visual framework of Bayesian optimization (BO) for HP selection.

This is repeated up to a predetermined number of times to fine-tune the validation set and produce optimized parameters that improve categorization. An illustration of the process is shown in Figure 5. This figure shows that the data has been selected at the initial stage and then defines the HP that needs to be optimized. After that, define the acquisition function that further executes the number of trials and choose the best HP to train the proposed architecture. The BO returned the selected HP as listed in Table 2.

**Table 2:** Optimized hyper parameters for network training using BO.

HP name	Range	Value
Initial learning rate	0.01–0.999	0.00167
Epochs	1–100	52
Momentum	0–1	0.669
Mini-batch size	16–256	128
L2-regularization factor	0.01–1	0.0045

The utilized acquisition function is the expected improvement (EI). EI calculates the anticipated level of enhancement achievable when exploring the vicinity around the current optimal value. If the function value’s apparent improvement is less than what was predicted after the process, indicating that a local optimum may exist, the algorithm will look into other areas of the domain to determine the ideal value. The quantification of improvement (L) hinges on evaluating the disparity between the function value at the sampled point and the prevailing optimal value. If the function value at the sampled point is below the current optimum value, the improvement is set to 0.


(1) $$L\left(e\right)=\max\left\{0,f_{t+1}\left(e\right)-f\left(e\right)\right\}$$

The goal is to optimize the expected improvement (EI) by maximizing it with regard to the current optimal value (*f*) through the implementation of the expected improvement (EI) optimization approach.


(2) $$\arg\max E\left[I\left(e\right)\right]=\arg\max E\left(\max\left\{0,f_{t+1}\left(e\right)-f\left(e^{+}\right)\right\}\right)$$

When $f_{t+1}\left(e\right)-f\left(e^{+}\right)\geq 0,$ the distribution $f_{t+1}\left(e\right)$ of follows a normal distribution with the mean $\mu \left(e\right)$, and the standard deviation, $\sigma^{2}\left(e\right)\text{.}$ consequently, the distribution of the random variable L is also a normal distribution, with the mean $\mu \left(e\right)-f\left(e^{+}\right)$ and standard deviation both being equal to $\sigma^{2}\left(e\right)$. The probability density function of I is


(3) $$f\left(I\right)=\frac{1}{\sqrt{2\pi \sigma \left(e\right)}}\exp\left(-\frac{{\left(\mu \left(e\right)-f\left(e^{+}\right)-L\right)}^{2}}{2\sigma^{2}\left(e\right)}\right),I\geq 0$$

The *E*(*I*) function is utilized to compute the expected degree of improvement attainable by exploring the vicinity around the current optimal value. If the observed increase in the function value during algorithm execution falls short of the expected value, it indicates that the current optimal value point might represent a local optimum. In such cases, the algorithm will continue to search for the optimal value point in alternative domain locations. The formulation of expected improvement is outlined as follows:


(4) $$\begin{array}{c} E\left(I\right)\int_{\infty }^{\infty }\text{If}\left(I\right)\;eI=\int_{I=0}^{I=\infty }I\frac{1}{\sqrt{2\pi \sigma \left(e\right)}}\exp\left(-\frac{{\left(\mu \left(e\right)-f\left(e\right)-I\right)}^{2}}{2\sigma^{2}\left(e\right)}\right)eI\\ =\sigma \left(e\right)\left[w\emptyset \left(\omega \right)+\emptyset \left(w\right)\right] \end{array}$$

where


(5) $$w=\frac{\mu \left(e\right)-f\left(e\right)\;}{\sigma \left(e\right)}$$

The anticipated improvement (*I*) is expressed by Eq. (4), encapsulating the definition of the expected improvement (EI) function. The stopping criterion for Bayesian optimization (BO) was set at 30 iterations. Following the completion of 30 iterations, the model with the minimum error among all iterations was chosen for the feature extraction process.

#### 2.3.2 Features extraction

Deep features are extracted from the self-attention activation of the trained deep learning architecture. In this layer, a feature vector of size *N* × 2048 is obtained. However, examination reveals that the features that were collected contain redundant data that can be optimized by utilizing a moth-flame optimization technique inspired by nature, specifically modified search-based moth-flame optimization.

### 2.4 Algorithm for modified search-based moth-flame optimization

This work selects the moth-flame optimization algorithm due to the improved performance of a few existing methods such as GSA, PSO, BA, FA, FPA, and a few more [see (28)]. The key advantage of MFO over other algorithms is its ability to solve difficult issues with confined and unknown search spaces (29). More than 160,000 various kinds of moths have been identified in nature; their life cycles are similar to those of butterflies (29). The most remarkable aspect of moth life is their unique night navigational system. They have developed the ability to use moonlight to fly at night.

Moreover, moths employ transverse orientation as a navigation strategy. By consistently adjusting their angle relative to the moon, moths efficiently navigate and cover substantial distances in a straight line. This approach ensures straight-line flight since the moon remains far from the moth. Interestingly, humans can adopt a similar navigation method. For instance, when the moon is situated in the southern sky, a person heading east can maintain a straight path by keeping the moon on their left side. While moths utilize a spiraling motion around lights, diverging from the straight-line flight, this behavior is a result of the transverse orientation’s effectiveness with distant light sources like moonlight. In contrast, moths attempt to maintain a consistent angle with the light source in artificial human-generated light, leading them to travel in spiraling patterns around such lights.

There are three basic steps in the MFO algorithm. We have updated each phase with mathematical formulation. In the first phase, the initial population is defined as follows:


(6) $$Z=\left[\begin{array}{ccccc} z_{1,1} & z_{1,2} & \cdots & \cdots & z_{1,x}\\ z_{2,1} & z_{2,2} & \cdots & \cdots & z_{2,x}\\ \vdots & z_{i,j} & \vdots & \vdots & \vdots \\ z_{y,1} & z_{y,2} & \cdots & \cdots & z_{y,x} \end{array}\right]$$

where x represents the number of dimensions in the solution space, and y is the number of moths. These initial populations have been sorted into a descending order as follows:


(7) $$\tilde{Z}=\left[\begin{array}{ccccc} {\tilde{z}}_{1,1} & {\tilde{z}}_{1,2} & \cdots & \cdots & {\tilde{z}}_{1,x}\\ {\tilde{z}}_{2,1} & {\tilde{z}}_{2,2} & \cdots & \cdots & {\tilde{z}}_{2,x}\\ \vdots & {\tilde{z}}_{i,j} & \vdots & \vdots & \vdots \\ {\tilde{z}}_{y,1} & {\tilde{z}}_{y,2} & \cdots & \cdots & {\tilde{z}}_{y,x} \end{array}\right]$$

where ${\tilde{z}}_{i,j}$ denotes the sorted population of ith moth and jth dimension. Please note the initial matrix should be Z as defined in Eq. (6). The main purpose of this update is to maintain the best populations in the descending order for selection in the next step with minimum computational time. Additionally, an array is memorized that contains the following fitness values for each moth, where $L\in \tilde{Z}$:


(8) $$L=\left[\begin{array}{c} L_{1}\\ L_{2}\\ \vdots \\ L_{y} \end{array}\right]$$

Flames make up the remaining components of the MFO algorithm. The flames in x -dimensional space are displayed in the matrix below, along with a vector representing their fitness function:


(9) $$M=\left[\begin{array}{ccccc} M_{1,1} & M_{1,2} & \cdots & \cdots & M_{1,x}\\ M_{2,1} & M_{2,2} & \cdots & \cdots & M_{2,x}\\ \vdots & \vdots & \vdots & \vdots & \vdots \\ M_{y,1} & M_{y,2} & \cdots & \cdots & M_{y,x} \end{array}\right]$$

(10) $$F=\left[\begin{array}{c} F_{1}\\ F_{2}\\ \vdots \\ F_{y} \end{array}\right]$$

MFO uses three different functions to converge towards the global optimum in optimization problems. These functions have the following definitions:


(11) $$M_{F}=\left(M,V,S\right)$$

where M denotes the moths’ initial, haphazard positions, Moth movement in the search area is denoted by the letters N, and search completion is denoted by S. This function, denoted by *M*, produces the fitness value and random populations of moths.


(12) $$M:\psi \to \left(M,FM\right)$$

The moth’s travel to search space is denoted by V and defined as follows:


(13) V:ψ→ψ

The function S represents the stop criteria, and it is defined as follows:


(14) $$S:M\to \left(1,0\right),\text{where}\;1\in \text{True}\;\text{and}\;0\in \text{False}$$

This process is processed through a fitness function defined in Eq. (15).


(15) $$Ft=\left|\frac{f\left(\psi_{\text{best}}\right)}{f\left(\psi_{i}^{k}\right)}\right|$$

The exploitation phase focuses on improving the MFO algorithm’s utilization (updating the moth’s positions in n different places throughout the search area may reduce the likelihood of exploitation of the most promising solutions). Based on the fitness value, it can define whether the best position moth is selected. The updated position is defined as follows:


(16) $$\psi_{i}^{k}=d_{i}^{k-1}\;e^{bt}\cos\left(2\pi t\right)+Ft.Ft_{i}^{k-1}+\left(1-Ft\right).\psi_{\text{best}}$$

where $d_{i}^{k-1}$ denotes the distance among selected flames, and Ft is a fitness function. The final selection is usually performed by employing a threshold value of 0.5, but in this work, we consider the mean value of the selected features in each iteration and the cost function is defined as:


(17) $$\psi_{\text{cost}}=\tau_{\alpha }\times \partial_{\text{error}}+\tau_{\beta }\times \left(\frac{\text{num\_feat}}{\text{max\_feat}}\right)$$

(17a) $$\partial_{\text{error}}=1-\varphi_{\text{accuracy}}$$

In the above equation, $\tau_{\alpha }$ and $\tau_{\beta }$ denotes the coefficients and the values of $\varphi_{\alpha }\;\text{is}\;0.99\;\text{and}\;\varphi_{\beta }\;\text{is}\;0.01$, $\psi_{\text{cost}}$ presented the cost function and $\varphi_{\text{accuracy}}$ presented the accuracy obtained from the fitness function. The selected optimized features are finally classified using neural network classifiers for the final classification.

Where $d_{i}^{k-1}$ denotes the distance among selected flames, and Ft is a fitness function. The final selection is usually performed by employing a threshold value of 0.5, but in this work, we consider the mean value of the selected features in each iteration. The resulting feature vector for the fetal dataset is of size *N* × 2048, and for the Brain dataset, it is *N* × 996.

The selected optimized features are finally classified using neural network classifiers for the final classification.

## 3 Results and analysis

The experimental process of this work has been discussed in this section using tabular information, visual graphs, and confusion matrices. In addition, a comparison is also conducted to show the effectiveness of the proposed method.

### 3.1 Experimental setup

A publicly available dataset has been utilized, as discussed in section 2.1. The selected datasets were in RGB nature and the whole datasets were partitioned into 50:50 ratio. The 50% is used for training process and remaining 50% data is utilized for testing phase. The entire experimental process has been conducted utilizing a 10-fold cross-validation approach. The entire experimental process has been conducted utilizing a 10-fold cross-validation approach. The proposed CNN architecture has been trained with optimized HP using the common maternal fetal and fetal brain plane datasets, as presented in Table 2. During the validation phase, several neural networks were employed, and the best classifier was chosen based on the calculated performance measures such as accuracy, processing time, sensitivity rate, precision rate, the number of observations, Kappa, MCC, FNR, and *F*1-score. The simulations of this work have been conducted utilizing MATLAB 2023a on a workstation with 256 GB of RAM and 12GB graphics card RTX 3000.

### 3.2 Performance measures and experiments

The evaluation of the classification model for fetal brain and general fetal characteristics was based on a set of performance evaluation measures, as presented in Table 3. True positive (TP) denotes the rate of accurately predicted positive instances, while true negative (TN) represents the accuracy of the predicted negative class. False positive (FP) signifies an incorrect prediction of the positive rate, and false negative (FN) indicates an erroneous prediction of the negative rate.

**Table 3:** Performance measures for the evaluation of the proposed framework.

Name	Formula
Precision	$\frac{\text{TP}}{\text{TP}+\text{TP}}$
*F*1-score	$\frac{2}{\frac{1}{\text{Sensitivity}}+\frac{1}{\text{Precision}}}$
Accuracy	$\frac{\text{TP}+\text{TN}}{\text{TP}+\text{TN}+\text{FP}+\text{FN}}$
Sensitivity	$\frac{\text{TP}}{\text{TP}+\text{FN}}$
False-negative-rate	$\frac{\text{FN}}{\text{FN}+\text{TP}}$
False positive-rate	$\frac{\text{FP}}{\text{FP}+\text{TN}}$
Area under curve	$\int T\text{PR}\;d\left(\text{FPR}\right)$
Recall rate	$\frac{\text{TP}}{\text{TP}+\text{FN}}$
Kappa	$\text{Kappa}\;\left(K\right)=\frac{\text{Po}-\text{Pe}}{1-\text{Pe}}$

### 3.3 Fetal brain dataset

**Figure 6:**
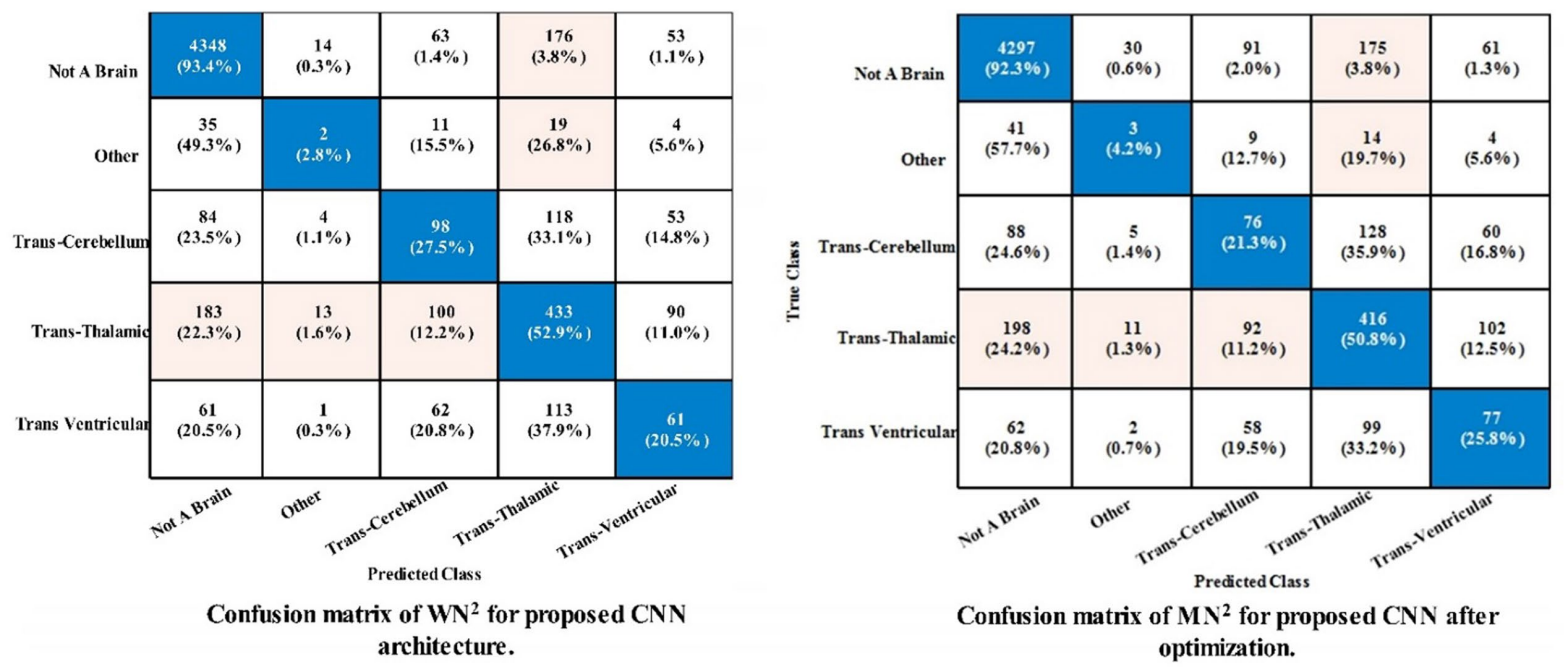
Confusion matrix of the proposed framework using fetal brain dataset.

Table 4 demonstrates the classification results of the proposed CNN architecture without feature optimization. The fetal brain dataset has been employed for the classification results. The maximum reported accuracy in this table is 79.7% for the WN^2^ classifier. For this classifier, the recall rate of 39.42%, the precision rate of 40.26%, *F*1 scores of 39.69%, and AUC values of 0.80. Moreover, the classifier’s confusion matrix is shown in Figure 6, which can be used to verify the calculated performance measures according to Table 3’s specifications. The rest of the classifiers also obtained 76.9% to 78.6% accuracy. Every classifier’s computational time is also recorded; the WN^2^ classifier has the lowest recorded computational time of 155.46 s.

**Table 4:** Classification results of proposed CNN architecture using ultrasound images (fetal brain).

Classifiers	Accuracy (%)	Sensitivity rate (%)	Precision rate (%)	Kappa	MCC	*F*1 score	AUC	Time complexity
N^3^	77.8	39.12	38.26	0.3053	0.3031	0.3864	0.73	1,528
MN^2^	78.6	39.08	38.96	0.3320	0.3091	0.3898	0.69	159.47
**WN** ^**2**^	**79.7**	**39.42**	**40.26**	**0.3663**	**0.3211**	**0.3969**	**0.80**	**155.46**
BN^2^	77.1	37.38	37.42	0.2847	0.2869	0.3729	0.73	1424.2
TN^2^	76.9	37.12	36.76	0.2781	0.2846	0.3690	0.74	1293.3

Bold indicate the best values.

The classification outcomes of the proposed framework using an improved optimization technique are displayed in Table 5. The best features are selected and obtained the highest accuracy value of 78.5% for MN^2^ classifier. Other parameters are also computed for this classifier, such as a recall rate of 38.88%, precision rate of 39.24%, *F*1 scores of 0.3903%, and AUC values of 0.70%. Moreover, Figure 6 depicts the MN^2^ confusion matrix. Through this figure, the computed measures of this classifier can be confirmed. Table 5 also lists the computational time for each classifier. There is an apparent reduction in time after the application of the optimization algorithm as compared to Table 4. The minimum noted time after this step is 115.14 s for WN^2^ classifier.

**Table 5:** Classification results of proposed architecture after employing improved optimization using brain fetal ultrasound images.

Classifier	Accuracy (%)	Sensitivity rate (%)	Precision rate (%)	Kappa	MCC	*F*1 Score	AUC	Time complexity
N^3^	76.6	37.12	36.64	0.2695	0.2815	0.3685	0.91	693.84
**MN** ^**2**^	**78.5**	**38.88**	**39.24**	**0.3295**	**0.3080**	**0.3903**	**0.70**	**133.09**
WN^2^	78.4	37.94	38.54	0.3265	0.3005	0.3816	0.79	115.14
BN^2^	77.2	37.36	37.24	0.2867	0.2866	0.3727	0.71	661.27
TN^2^	76.4	36.8	37.00	0.2634	0.2803	0.3682	0.73	618.06

Bold indicate the best values.

### 3.4 Common maternal fetal dataset results

**Figure 7:**
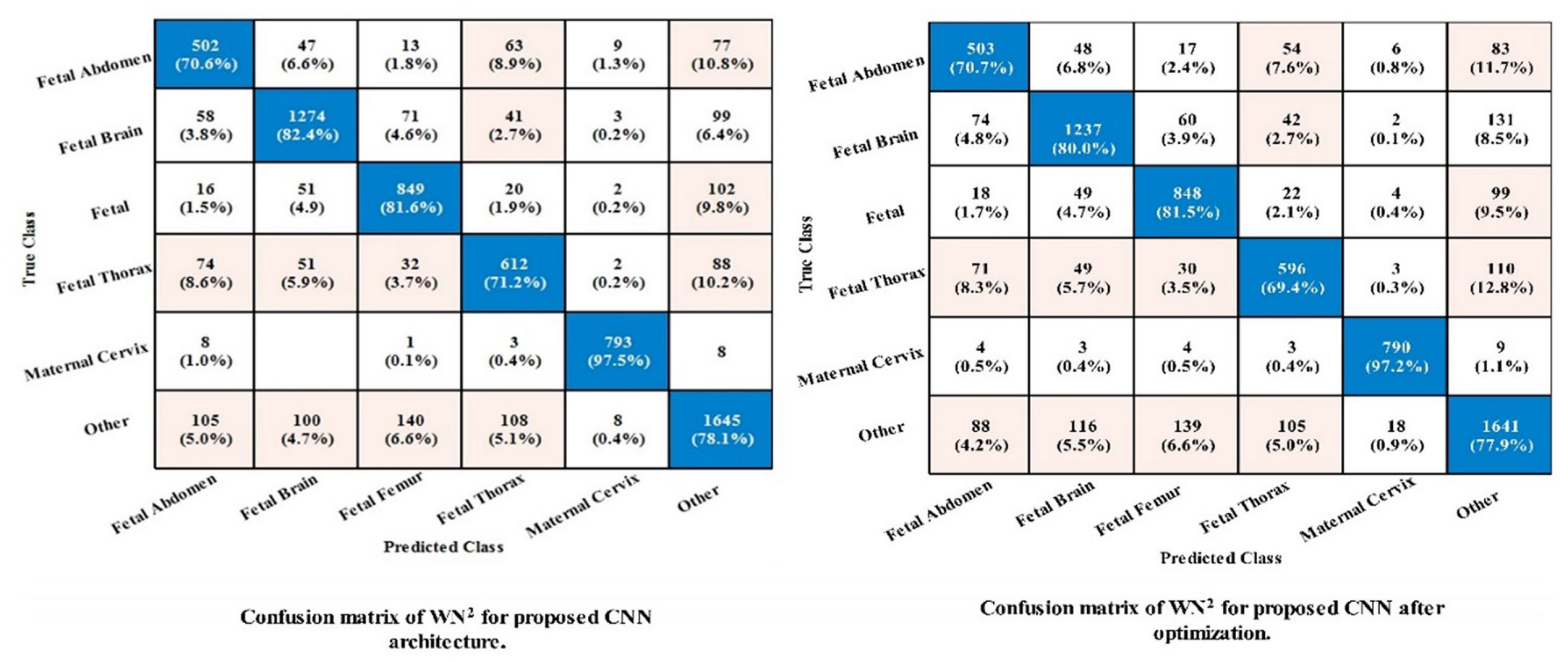
Confusion matrix of the proposed framework using common maternal fetal dataset.

Table 6 demonstrates the classification of proposed CNN architecture results for common maternal fetal dataset. The proposed architecture obtained a maximum accuracy of 79.8% for MN^2^. The recall rate of this classifier is 80.15%, a precision rate of 79.08%, *F*1 score of 0.795, and AUC value of 0.91, respectively. To further confirm the accuracy of each class’s prediction rate and computed performance metrics, refer to Figure 7, which shows the confusion matrix of this classifier. The computational time of each classifier is also noted, and the minimum time is 215.87 s for the WN^2^ classifier, whereas the highest reported time is 1476.1 (sec). The comparison is also performed with a few other classification methods, as given in this table, and the computed accuracy range is between 77.3% and 79.8%.

**Table 6:** Classification results of proposed bottleneck CNN architecture using common maternal fetal ultrasound planes.

Classifiers	Accuracy (%)	Sensitivity rate (%)	Precision rate (%)	Kappa	MCC	*F*1 score	AUC	FNR	Time complexity
N^3^	78.2	78.1	77.36	0.213	0.731	0.776	0.90	20.89	1476.1
MN^2^	79.8	80.15	79.08	0.274	0.754	0.795	0.91	19.85	222.55
**WN** ^**2**^	**80.2**	**80.23**	**79.53**	**0.287**	**0.757**	**0.798**	**0.93**	**19.77**	**215.87**
BN^2^	77.7	77.78	76.85	0.195	0.726	0.772	0.89	22.22	1118.7
TN^2^	77.3	77.51	76.48	0.182	0.722	0.769	0.89	22.49	1142.5

Bold indicate the best values.

The noted accuracy is better, but the computational time is inefficient; therefore, we employed the optimization algorithm, and Table 7 provides a full breakdown of the outcomes. In this table, the best obtained accuracy of 79.4% for WN^2^, which is minor decreased than the original architecture accuracy but time is 81.438 (sec). The accuracy of this classifier can be confirmed by a Figure 7 (confusion matrix). In this figure, each class correct prediction rate is given diagonally. The previous time of WN^2^ was 215.87 (sec); hence, the time of the proposed architecture after employing the optimization algorithm is reduced which is a strength of this work.

**Table 7:** Classification results of proposed architecture after employing improved optimization using common maternal fetal ultrasound images.

Classifiers	Accuracy (%)	Sensitivity rate (%)	Precision rate (%)	Kappa	MCC	*F*1 score	AUC	FNR	Time complexity
N^3^	75.7	75.88	75.05	0.125	0.704	0.754	0.9	24.12	270.06
MN^2^	77.7	77.86	77.025	0.197	0.728	0.774	0.91	22.14	89.497
**WN** ^**2**^	**79.4**	**79.45**	**78.95**	**0.256**	**0.748**	**0.791**	**0.93**	**20.55**	**81.438**
BN^2^	75.1	75.31	74.53	0.104	0.6971	0.7483	0.88	24.69	241.25
TN^2^	75.4	75.83	75	0.1148	0.7028	0.7536	0.89	24.17	243.83

Bold indicate the best values.

### 3.5 Comparison with SOTA

**Figure 8:**
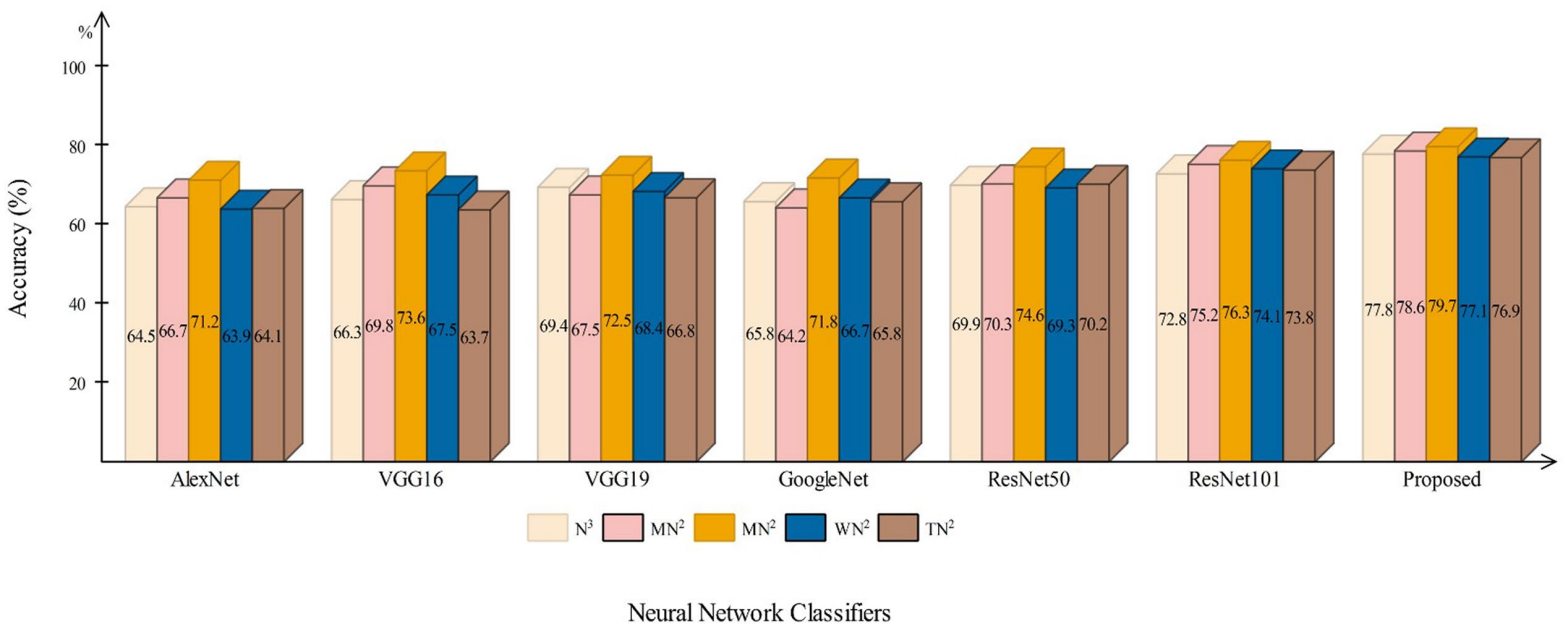
A comparison among several pre-trained neural nets with proposed architecture using brain fetal ultrasound images.

**Figure 9:**
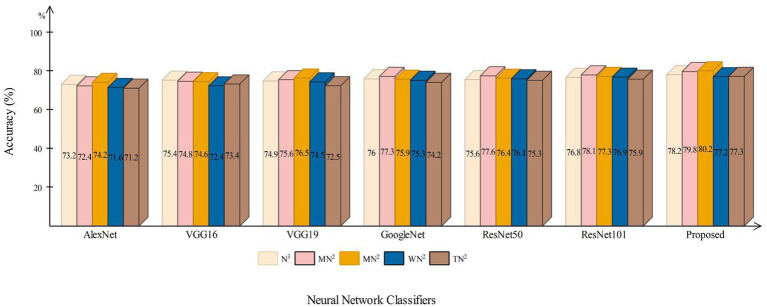
A comparison among several pre-trained neural nets with proposed architecture using common maternal fetal ultrasound images.

This section presents a detailed comparison between the proposed framework and several state-of-the-art (SOTA) approaches is given in this section. The comparison is carried out in two stages. In the first step, several pre-trained neural networks were chosen and trained on the same datasets (fetal brain and common maternal-fetal). Figure 8 compares several pre-trained neural nets with the proposed CNN architecture for the brain fetal dataset. The proposed architecture accuracy is improved, and almost a 2% difference is noted in accuracy. After the proposed architecture, the ResNet models show improved accuracy. Figure 9 compares several pre-trained neural nets using the common maternal fetal dataset with the proposed architecture. This figure shows that the maximum obtained accuracy by other neural nets is 77.3% by ResNet101, whereas the proposed architecture shows an improved accuracy of 80.2%.

A comparison among improved MFO is also conducted with original MFO and a few other nature-inspired optimization algorithms. As described in Table 8, the proposed architecture obtained the highest accuracy of 78.5% and 79.4% using the IMFO algorithm for brain fetal planes and common maternal fetal planes, respectively. The proposed architecture obtained 73.8% and 74.6% accuracy for GA-based feature selection. For PSO-based feature selection, an accuracy of 74.9% and 75.1% were obtained, respectively. The paired proposed architecture with the Whale optimization algorithm (WOA) has achieved 73.3% and 75.6% accuracy, respectively. The paired firefly algorithm improved the accuracy value by 77.8%. The second highest accuracy for both datasets is 76.9% and 78.1% by the original moth flame optimization algorithm. Hence, this table shows that the proposed architecture performed well with IMFO. In addition, a computational time of each pair has been noted and it is observed that the IMFO algorithm time is minimum than all other combinations.

**Table 8:** Comparison of the proposed architecture with several other state-of-the-art optimization algorithms.

Methods	Brain fetal planes	Time (sec)	Common maternal fetal planes	Time (sec)
**Proposed** +**IMFO**	**78.5**	**133.09**	**79.4**	**81.438**
Proposed + GA (30)	73.8	141.773	74.6	92.990
Proposed + PSO (31)	74.9	146.328	75.1	91.643
Proposed + WOA	73.3	153.673	75.6	103.563
Proposed + BCO	74.7	140.430	76.3	111.336
Proposed + FA	75.8	141.556	77.8	95.245
Proposed + ACO	75.2	136.564	76.5	110.511
Proposed + MFO	76.9	151.995	78.1	104.264

Bold indicate the best values.

## 4 Conclusion

An automated deep learning architecture has been proposed in this work to classify brain and common maternal fetal plane classification. A new deep learning architecture has been designed based on bottleneck residual blocks. The proposed architecture consists of 82 layers with three blocks, including 2 highway paths and one skip connection. The hyper parameters have been chosen through Bayesian optimization (BO) rather than manual initialization. The proposed architecture obtained an accuracy of 78.5% and 79.4% for brain and common maternal fetal images. However, a high computational time is noted during the classification process; therefore, We implemented an enhanced optimization algorithm for feature selection, resulting in a substantial (100%) reduction in computational time. After employing the optimization algorithm, a minor change occurred in the accuracy and precision value. In addition, we compared the proposed optimization algorithm accuracy with several SOTA techniques, and the IMFO algorithm showed a better performance.

There are few dark sides of this work: (i) imbalanced dataset is a problem for the training of a deep learning model; (ii) irrelevant information extraction from the deep layer. In future, a problem of imbalance dataset has been resolved and proposed Self-attention mechanism architecture with an inverted bottleneck block to reduce computational time and irrelevant information extraction.

## Data availability statement

The original contributions presented in the study are included in the article/supplementary material, further inquiries can be directed to the corresponding authors.

## Author contributions

FR: Conceptualization, Methodology, Software, Writing – original draft. MK: Methodology, Software, Supervision, Writing – original draft, Writing – review & editing. AB: Data curation, Formal analysis, Investigation, Software, Writing – review & editing. KJ: Investigation, Methodology, Software, Writing – original draft, Writing – review & editing. AH: Conceptualization, Data curation, Resources, Software, Validation, Writing – original draft. AA: Formal analysis, Methodology, Project administration, Resources, Visualization, Writing – review & editing. NA: Conceptualization, Investigation, Methodology, Project administration, Validation, Visualization, Writing – review & editing. AM: Funding acquisition, Methodology, Visualization, Writing – original draft, Writing – review & editing.
